# Supplementary material for: Picosecond orientational dynamics of water in living cells
Source: Nat Commun. 2017 Oct 12;8:904. doi: 10.1038/s41467-017-00858-0 (PMC5714959; doi:10.1038/s41467-017-00858-0)
Supplement: Supplementary file 1 — Supplementary Information [file 41467_2017_858_MOESM1_ESM.pdf]

SUPPLEMENTARY FIGURE

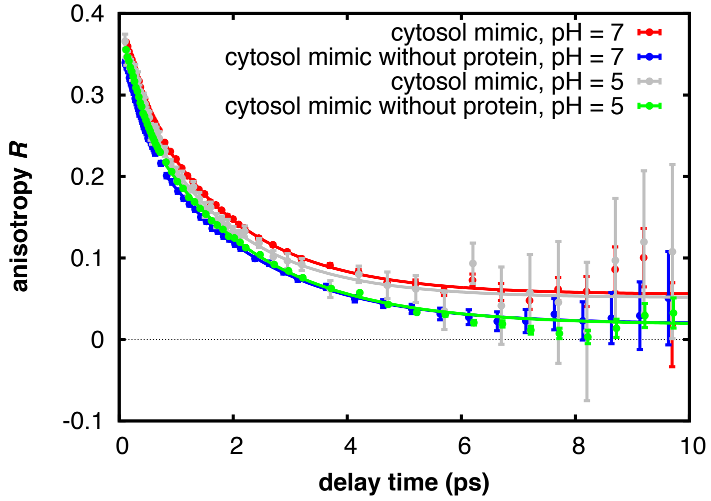

Supplementary Figure 1. Transient anisotropy of the OD-stretch mode (2508 cm^{-1}) of HDO water in different cytosol mimics: with and without protein, and at two pH values. The curves are least-squares fits to single-exponential decays with a residual offset (see Supplementary Table 2 for the fit parameters).

SUPPLEMENTARY TABLES

Supplementary Table 1. Compositions of cytosol mimic solutions with different pH values. Of both solutions two versions were made: one with 30% BSA protein and one without.

**pH**	**5.0**	**7.0**
$H_2PO_4^{-}$/ HPO_4^{2-}	50 mM	50 mM
SO_4^{2-}	2.5 mM	2.5 mM
glutamate	250 mM	250 mM
Ca^{2+}	0.5 mM	0.5 mM
Mg^{2+}	2 mM	2 mM
Na^{+}	20 mM	20 mM
K^{+}	272 mM	339 mM

Supplementary Table 2. Results of the single-exponential least-squares fits to the vibrational anisotropy. The error bars are 2σ. Rates $1/\tau_{or}^{IR}$ and residuals $R_{residual}$ were obtained from fits to the presented in article Figure 2 and Supplementary Figure 1, with a fit function $Ae^{-t/\tau_{or}^{IR}} + R_{residual}$, and with all fits starting at t = 0.8 ps to avoid contributions from the ND stretch mode, see Supplementary Discussion 1.

**Sample**	$1/\tau_{or}^{IR}$ **(ps^{-1})**	$R_{residual}$
neat water	0.46±0.01	0 (not fitted)
E. coli	0.54±0.04	0.053± 0.007
yeast	0.55± 0.04	0.051±0.006
spores	0.71± 0.09	0.085±0.010
cytosol mimic, with protein, pH = 7	0.59±0.03	0.055±0.004
cytosol mimic, no protein, pH = 7	0.51±0.02	0.018±0.004
cytosol mimic, with protein, pH = 5	0.62±0.1	0.051±0.014
cytosol mimic, no protein, pH = 5	0.51±0.03	0.018±0.006

Supplementary Table 3. Fit parameters obtained from fitting eq S1 in the Supplementary Discussion 2 to the experimental spectra of three organisms and cytosol mimic (pH 7, with protein): relaxation strengths, S_j, relaxation times, τ_j, and Cole-Cole parameters, α_j, for the δ-relaxation (j = δ) and water (j = water), together with the conductivity of the samples, κ. Errors correspond to the standard deviation within at least 3 independent experiments.

	ε_1	τ_δ/ps	α_δ	ε_2	τ_{water}/ps	α_{water}	ε_∞	κ/Sm^{-1}
E. Coli	52.2±0.8	104±14	0.00±0.01	50.3±0.7	8.4±0.2	0.05±0.02	5.3±0.4	0.52±0.1
spores	40.6±5.7	2000±1000	0.01±0.01	37.6±0.3	8.9±0.1	0.05±0.01	5.1±0.1	0.05±0.07
yeast	62.4±0.6	109±2	0.07±0.06	52.3±1.3	9.1±0.2	0.04±0.01	5.5±0.2	0.79±0.01
cyt. mimic	64.0±0.9	97±3	0.14±0.04	49.1±0.5	8.9±0.2	0.03±0.01	5.6±0.2	1.51±0.02

SUPPLEMENTARY DISCUSSIONS

Supplementary Discussion 1. Contribution of ND and non-water-OD groups to the infrared absorbance and anisotropy

In the cells, the infrared absorption and anisotropy at the OD-stretch frequency (~2500 cm^{-1}) both originate predominantly from the OD-stretch mode of the water (HDO) molecules. To estimate the maximum contribution due to ND and non-water-OD groups (formed by H/D exchange with HDO) to the absorption and anisotropy, we first determine the intracellular concentration of NH and non-water-OH groups based on the known constitution of the spores,[1] E. coli,[2] and yeast,[3–5] as detailed below. In these listings the contributions of NH groups are underlined.

E. coli

Total mass cell: m(cellTot) = 9.57*10(-13) g

Water content: m(H2O) = 0.7*m(cellTot) = 6.7*10(-13) g

Dry weight: m(dry) = 0.3*m(cellTot) = 2.87*10(-13) g

Protein: 55% of dry weight

m(prot) = 156*10(-15) g → n(NH,backbone) = n(AA) = 1.42*10(-15) mol

(average mass amino acid = 110 g/mol)

Amino-acid side groups (based on known amino-acid composition of the cell):

Ser: 5.8*10(-17) * 1 mol

Arg: 8.0*10(-17) * 5 mol

Hist: 2.5*10(-17) * 1

Thr: 6.9*10(-17) * 1

Asn: 6.5*10(-17) * 2

Gln: 7.1*10(-17) * 2

Tyr: 3.7*10(-17) * 1

------------------------- +

n(OH) = 1.6*10(-16) mol

n(NH) = 6.9*10(-16) mol

DNA:

n(NH) per nucleotide: G=3,C=2,T=1,A=2 on average 2

n(bases) = 4.6*10(6) bp = 9.2*10(6) bases/nuc →

n(NH) = 9.2*10(6)*2 = 18.4*10(6) = 3.0*10(-17) mol

RNA: 20.7% dry weight

Avg m(nucleotide) = 324.3 g/mol

m(RNA) = 0.207*2.87*10(-13) = 58*10(-15) g → n(nuc)=1.79*10(-16)

n(NH) = 2*1.79*10(-16)= 3.6*10(-16) mol

Liposaccharides

n/cell = 1430000 molecules → n(OH)=39*1430000=55770000=9.3*10(-17) mol

Peptidoglycan

n/cell = 904 monomers → n(OH) = 904*5 = 4520 = negligible

Polyamines

-Putrescine: n/cell = 5600000 molecules → n(NH) = 2*5600000 = 1.85*10(-17) mol

-Spermidine: n/cell = 1100000 molecules → n(NH) = 3*1100000 = 0.55*10(-17) mol

Water OH groups: n(OH) = 2*(6.7*10(-13)/18) = 7.4*10(-14) mol

Total number of NH and OH groups =

(7.4+0.142+0.016+0.069+0.003+0.036+0.0093+0.00185+0.00055)*10(-14) = 7.67*10(-14) mol

Yeast

Total mass cell: m(cellTot) = 60*10(-12) g

Water content: m(H2O) = 0.65*m(cellTot) = 39*10(-12) g

Dry weight: m(dry) = 0.35*m(cellTot) = 21*10(-12) g

Protein: 39.6% dry weight

n(NH,backbone) = n(AA) = 21*10(-12)*0.396/110 = 7.56*10(-14) mol

Amino-acid side groups (based on known amino-acid composition of the cell; in Ref. 2 used for this table only the total fractions of Glu+Gln and of Asp+Asn are given. To obtain the separate

contributions, we used the abundances given in Table I of Supplementary Reference 6):

Ser: 5.3% → 0.4*10(-14) mol

Arg 3.8% → 1.4*10(-14) mol

Hist: 1.9% → 0.14*10(-14) mol

Thr: 5.5% → 0.41*10(-14) mol

Tyr: 1.9% → 0.14*10(-14) mol

Asn: 2.3% → 0.17*10(-14) mol

Gln: 3.9% → 0.29*10(-14) mol

-------------------------- +

n(OH) = 0.95*10(-14) mol

n(NH) = 2.0*10(-14) mol

RNA: 9% dry weight

m = 1.89*10(-12) → n(NH) = 1.89*10(-12)/324.3*2 = 1.31*10(-14) mol

Cell wall: 24.5% dry weight

m = 5.1*10(-12) → n(OH) = 5.1*10(-12)/(0.1*627+0.9*180)=2.3*10(-14) mol

DNA: 1*10(-16) mol (negligible)

Water OH groups: n (OH) = 39*10(-12)*2/18 = 4.33*10(-12) mol

Total number of NH and OH groups = (4.33+0.0756+0.0095+0.02+0.0131+0.023)*10(-12) = 4.47*10(-12) mol

Spores

Total mass cell: m(cellTot) = 7.02*10(-13) g

Water content: m(H2O) = 0.4*m(cellTot) = 2.81*10(-13) g

Dry weight: m(dry) = 0.6*m(cellTot) = 4.21*10(-13) g

Protein: 76% dry weight

m(prot) = 0.76*4.21*10(-13)=3.20*10(-13) →

n(AA) = 3.20*10(-13)/110=2.90*10(-15) mol → n(NHbackbone) = 2.90*10(-15) mol

Amino-acid side groups (based on known amino-acid composition of the cell; in Ref. 1 used for this table only the total fractions of Glu+Gln and of Asp+Asn are given. To obtain the separate contributions, we used the abundances given in Table I of Supplementary Reference 6):

Ser: 4% → 1.60*10(-16) mol

Arg: 3% → 5*0.73*10(-16) mol

Hist: 3% → 0.815*10(-16) mol

Thr: 5% → 1.77*10(-16) mol

Asn: 0.7% → 0.20*10(-16) mol

Gln: 1.3% → 0.37*10(-16) mol

Tyr: 6% → 1.74*10(-16) mol

-------------------------- +

Total: 10.14*10(-16) mol

n(OH) = 5.11*10(-16) mol

n(NH) = 5.03*10(-16) mol

Carbohydrate: 4%

n(monomers) = 0.04*4.21*10(-13)/504=3.3*10(-17) →

n(OH) = 3*3.3*10(-17) = 9.9*10(-17)

DNA: n(NH) =1.1*10(-19) = negligible

RNA: n(NH) = 1.498*10(-18) = negligible

Water OH groups: n(OH) = 2.81*10(-13)*2/18 = 3.12*10(-14) mol

Total number of NH and OH groups =

(3.12+0.29+0.1014+0.0099)*10(-14) = 3.52*10(-14) mol

From these numbers we conclude that the total fraction of ND and non-water-OD groups is 3.5% *E. coli*, 3.1% in yeast, and 11% in the bacterial spores; in all three cases most of this is ND. The remainder of the deuterons is present as HDO.

IR absorbance at 2500 cm^{-1}. The extinction coefficient of the protein-backbone ND-stretch mode (the dominant contribution to the non-HDO absorption) at 2500 cm^{-1} is smaller than

that of the OD-stretch mode of HDO (as we determined by comparing the IR spectra of dilute HDO:H_2O and NMA-d:NMA solutions of known composition). Hence, the contribution of ND and non-water-OD groups to the absorption at 2500 cm^{-1} is somewhat smaller than their molar fractions given above, so we can conclude that in the spores ~90% of the absorption is due to water, and in *E. coli* and yeast ~97%.

IR anisotropy at 2500 cm^{-1}. The contribution of the ND- and non-water-OD groups to the vibrational anisotropy will be approximately proportional to their abundances (for the ND groups slightly lower than their abundance, because of the smaller extinction coefficient of these groups at 2500 cm^{-1}; this effect enters quadratically in the anisotropy, which is measured in a nonlinear pump-probe experiment). In all samples this contribution is small (<4%) except for the bacterial spores, mostly due to ND-groups in the protein backbone, which constitute 8% of the total amount of OD/ND groups present in the spores. However, the ND-stretch mode of an amide group has a much shorter excited-state lifetime (T_1 = 0.58 ps for the amide A mode, ~0.7 ps for NH_2 groups)[7,8] than the OD-stretch mode of HDO (T_1 = 1.8 ps),[9] so that the contribution of the ND-groups decreases rapidly with increasing pump-probe delay. In particular, it can be shown that in the case of several molecular species contributing to the anisotropy, the total delay-dependent anisotropy *R(t)* can be written as (see the Supporting Information of Supplementary Reference 10):

$$R(t) = \sum_i c_i(t) R_i(t),$$

where i runs over all species present, each with their own anisotropy decay function $R_i(t)$, and where

$$c_i(t) = p_i e^{-t/T_{1,i}} / \sum_i p_i e^{-t/T_{1,i}},$$

with p_i and $T_{1,i}$ the abundance and excited-state lifetime of each species, and where we assume for simplicity that the extinction coefficients of all species are the same (taking into account the lower ND-stretch extinction coefficient will render the ND contribution even smaller). Using these equations it is easily shown that the contribution of the amide ND groups decreases from 8% at t = 0 to ~0.2% at t = 3 ps and even less for longer delay times, rendering this contribution negli-

gible for sufficiently long delay time. For this reason, we start our least-squares fits to the anisotropy decays at $t = 0.8$ ps.

Supplementary Discussion 2. Dielectric-relaxation spectroscopy

We measured complex permittivity spectra as a function of field frequency, ν, for the three organisms and the cytosol mimic at $0.76 \leq \nu/\text{GHz} \leq 70$. Complex permittivity spectra, $\hat{\varepsilon}(\nu) = \varepsilon'(\nu) - i\varepsilon''(\nu)$ were measured using a frequency domain reflectometer based on an Anritsu Vector Star MS4647A vector network analyzer with an open ended coaxial probe based on 1.85 nm coaxial connectors.[11–13] To calibrate for instrumental errors in directivity, source match, and frequency response[12] we used air, water[14] and conductive silver paint (short) as references. All measurements were performed at 23±1°C. For measurements of the organisms, small amounts of the samples were incrementally applied to the coaxial probe. At a total volume of ~ 0.1 mL the scattering parameter (recorded by vector network analyzer) plateaued upon addition of additional sample volume and these data were used for further analysis. Dielectric permittivity spectra were then calculated from the recorded scattering parameters using the model for the complex admittance reported by Blackham.[11,12]

To extract the contributions due to the collective relaxation of water centered at ~ 20GHz and the lower frequency relaxation due to interfacial polarizations, we fit a combination of two Cole-Cole type equations to the experimental spectra:

$$\hat{\varepsilon}(\nu) = \frac{\varepsilon_1 - \varepsilon_2}{1 + (i2\pi\nu\tau_\delta)^{(1-\alpha_\delta)}} + \frac{\varepsilon_2 - \varepsilon_\infty}{1 + (i2\pi\nu\tau_{\text{water}})^{(1-\alpha_{\text{water}})}} + \varepsilon_\infty + \frac{\kappa}{2\pi i\nu\varepsilon_0} \tag{S1}$$

Where the first two terms represent the Cole-Cole relaxations[15] with relaxation times, τ_j, and the Cole Cole parameter, α_j, which accounts for a symmetric broadening of the relaxation mode (with respect to a Debye type relaxation).[15] ε_j are the limiting permittivities of each relaxation, with the limiting permittivity at infinite frequencies, ε_∞, subsuming all polarizations above the frequencies of the present study. The last term in eq S1 accounts for Ohmic losses originating from the samples conductivity, where κ is the electrical (d.c.) conductivity. ε_0 is the permittivity of free space. The parameters obtained from fitting eq S1 to the spectra of the three organisms are summarized in Table S3.

The value of τ_{water} represents the collective relaxation time of the three dimensional hydrogen-bonded network of water, $\tau_{\text{or}}^{\text{DRS}}$, as discussed in the main text. The dielectric strength of the water relaxation $S_{\text{water}} = \varepsilon_2 - \varepsilon_\infty$ can be directly related to the molar concentration of water using e.g. the Cavell equation.[16] Hence, the ratio $S_{\text{water}}/S_{\text{water,neat}}$ provides an estimate for the volume fraction of bulk-like water

in the organisms ($S_{\text{water,neat}} = 72.68$).[14] Note that here we neglect local field effects,[15] as the high static dielectric constants (ε_1) of the present samples makes the local field correction to virtually cancel when considering the relaxation strength relative to neat water. Also the reduction of the dielectric strengths due to kinetic depolarization[17] is not accounted for, which may lead to a minor underestimation of the volume fractions for the samples with high conductivity (< 2% for cytosol mimic).

SUPPLEMENTARY REFERENCES

1. Gould, G. W. & Hurst, A. *The Bacterial Spore*. (Academic Press Inc, 1969).

2. Neidhardt, F. C. & Umbarger, E. *Escherichia coli and Salmonella: Cellular and Molecular Biology* (American Society of Microbiology Press, 1996).

3. White, J. Variation in water content of yeast cells caused by varying temperatures of growth and by other cultural conditions. *J. Inst. Brew.* **58,** 47–50 (1952).

4. Yamada, E. A. & Sgarbieri, V. C. Yeast (Saccharomyces cerevisiae) Protein Concentrate: Preparation, Chemical Composition, and Nutritional and Functional Properties. *J. Agric. Food Chem.* **53,** 3931–3936 (2005).

5. Aguilar-Uscanga, B. & François, J. M. A study of the yeast cell wall composition and structure in response to growth conditions and mode of cultivation. *Lett. Appl. Microbiol.* **37,** 268–74 (2003).

6. van Gulik, W. M. & Heijnen, J. J. A metabolic network stoichiometry analysis of microbial growth and product formation. *Biotechnol. Bioeng.* **48,** 681–698 (1995).

7. Rubtsov, I. V., Wang, J. & Hochstrasser, R. M. Vibrational Coupling between Amide-I and Amide-A Modes Revealed by Femtosecond Two Color Infrared Spectroscopy. *J. Phys. Chem. A* **107,** 3384–3396 (2003).

8. Rezus, Y. L. A. & Bakker, H. J. Effect of urea on the structural dynamics of water. *Proc. Natl. Acad. Sci.* **103,** 18417–18420 (2006).

9. Kropman, M. F., Nienhuys, H.-K., Woutersen, S. & Bakker, H. J. Vibrational Relaxation and Hydrogen-Bond Dynamics of HDO:H2O. *J. Phys. Chem. A* **105,** 4622–4626 (2001).

10. Dokter, A. M., Woutersen, S. & Bakker, H. J. Inhomogeneous dynamics in confined water nanodroplets. *Proc. Natl. Acad. Sci.* **103,** 15355–15358 (2006).

11. Ensing, W., Hunger, J., Ottosson, N. & Bakker, H. J. On the orientational mobility of water molecules in proton and sodium terminated nafion membranes. *J. Phys. Chem. C* **117,** 12930–12935 (2013).

12. Blackham, D. V. & Pollard, R. D. An improved technique for permittivity measurements using a coaxial probe. *IEEE Trans. Instrum. Meas.* **46,** 1093–1099 (1997).

13. Balos, V., Kim, H., Bonn, M. & Hunger, J. Dissecting Hofmeister Effects: Direct Anion-Amide Interactions Are Weaker than Cation-Amide Binding. *Angew. Chemie Int. Ed.* **55,** 8125–8128 (2016).

14. Fukasawa, T. *et al.* Relation between dielectric and low-frequency Raman spectra of hydrogen-bond liquids. *Phys. Rev. Lett.* **95,** 197802 (2005).

15. Böttcher, C. F. J. *Theory of Electric Polarization* (Elsevier, 1978).

16. Cavell, E. A. S., Knight, P. C. & Sheikh, M. A. Dielectric relaxation in non aqueous solutions. Part 2. Solutions of tri(n-butyl)ammonium picrate and iodide in polar solvents. *Trans. Faraday Soc.* **67,** 2225–2233 (1971).

17. Sega, M., Kantorovich, S. & Arnold, A. Kinetic dielectric decrement revisited: phenomenology of finite ion concentrations. *Phys. Chem. Chem. Phys.* **17,** 130–133 (2015).
